# A semi high-throughput method for real-time monitoring of curli producing *Salmonella* biofilms on air-solid interfaces

**DOI:** 10.1016/j.bioflm.2021.100060

**Published:** 2021-11-13

**Authors:** Ferdinand X. Choong, Smilla Huzell, Ming Rosenberg, Johannes A. Eckert, Madhu Nagaraj, Tianqi Zhang, Keira Melican, Daniel E. Otzen, Agneta Richter-Dahlfors

**Affiliations:** a AIMES - Center for the Advancement of Integrated Medical and Engineering Sciences at Karolinska Institutet and KTH Royal Institute of Technology, Stockholm, Sweden; b Department of Neuroscience, Karolinska Institutet, Stockholm, Sweden; c iNANO and Department of Molecular Biology and Genetics, Aarhus University, Aarhus, Denmark

**Keywords:** Optotracing, Biofilm, *Salmonella*, Curli, Real-time monitoring, Morphotyping

## Abstract

Biofilms enable bacteria to colonize numerous ecological niches. Bacteria within a biofilm are protected by the extracellular matrix (ECM), of which the fibril-forming amyloid protein curli and polysaccharide cellulose are major components in members of *Salmonella*, *Eschericha* and *Mycobacterium* genus. A shortage of real-time detection methods has limited our understanding of how ECM production contributes to biofilm formation and pathogenicity. Here we present optotracing as a new semi-high throughput method for dynamic monitoring of *Salmonella* biofilm growth on air-solid interfaces. We show how an optotracer with binding-induced fluorescence acts as a dynamic fluorescent reporter of curli expression during biofilm formation on agar. Using spectrophotometry and microscopic imaging of fluorescence, we analyse in real-time the development of the curli architecture in relation to bacterial cells. With exceptional spatial and temporal precision, this revealed a well-structured, non-uniform distribution of curli organised in distally projecting radial channel patterns. Dynamic monitoring of the biofilm also showed defined regions undergoing different growth phases. ECM structures were found to assemble in regions of late exponential growth phase, suggesting that ECM forms on site after bacteria colonize the surface. As the optotracer biofilm method expedites screening of curli production, providing exceptional spatial-temporal understanding of the surface-associated biofilm lifestyle, this method adds a new technique to further our understanding of bacterial biofilms.

## Highlights

• Design and evaluation of a method for real-time biofilm experimentation.
• Optotracing enables real-time monitoring of biofilm formation on solid supports.
• Definitive biofilm monitoring by selective tracking of ECM components.
• A method reducing the inherent biases of morphotyping.
• A semi-high throughput method increasing the ease and efficiency of biofilm detection.

## 1 Introduction

Biofilm is a multidimensional stage of life experienced by most bacterial species. The biofilm is composed of bacterial cells encased within a self-derived extracellular matrix (ECM) [1,2]. The ECM constitutes a major fraction of the biofilm dry mass, with only a minority attributed to bacterial cells [3]. While the ECM composition varies amongst microbes, each strain expresses a unique combination of components based on its genetic and metabolic capability. Some ECM components are considered ubiquitous, such as adhesins, amyloid proteins and polysaccharides [4]. For *Escherichia coli* (*E. coli*) and *Salmonella* species, the fibril-forming amyloid protein curli and the polysaccharide cellulose are expressed as major ECM components [5,6].

The biofilm community lifestyle offers bacteria protection from the action of antibiotics, the hosts’ immune responses, and environmental stresses, thereby enhancing the abilities to colonize biotic and abiotic surfaces [[7], [8], [9], [10], [11], [12], [13], [14]]. The fitness and virulence advantages are extensive, affecting porosity, density, water content, charge, absorption properties, hydrophobicity, and mechanical stability of the biofilm [15]. This is largely due to the high physical stability of ECM components. For example, curli fibrils resist extreme temperatures, pH, and denaturants, and only dissolve at high concentrations of formic acid [16,17]. Architectural studies of enterobacteria biofilm have shown complex but organised vertical layering of ECM components. In *E. coli* biofilm colonies on agar, growing cells and flagella are found at the bottom and outer rim of the macrocolony, wherein cells at the bottom are embedded in a mesh of flagella filaments [18]. Phosphoethanolamine-cellulose, a carbohydrate component in ECM of *E. coli* and *Salmonella*, strongly contributes to the overall architecture, enabling the development of vertical structures and conferring elasticity to the macrocolony [[19], [20], [21]]. Expression of ECM components is thus highly compartmentalised with deterministic effect on the thickness complexity of the biofilm [[18], [19], [20], [21]]. As a result, eradication of bacteria embedded within the ECM is considered difficult.

Across the many studies of biofilm ecology in various microenvironments, biofilm development is best understood in liquid perfused systems [22]. Yet in healthcare associated settings, the majority of surfaces that harbour microbes and facilitate transmission are solid surfaces. Experiments on agar plates have shown that the architecture of surface-associated biofilm communities is complex and adaptive, responding to environmental cues and chemical gradients that fluctuate over time [22]. Bacteria are thus exposed to different microenvironmental conditions when growing on solid surfaces compared to in liquid cultures. The bacterial gene expression pattern within biofilms on solid surfaces differs indeed from that of planktonic bacteria in liquid cultures [21,[23], [24], [25], [26], [27]]. Studies examining biofilm developmental stages on solid surfaces are however uncommon, since methods capable of continuous tracing of biofilm-specific components are scarce [28].

Optotracing was recently developed as a versatile method for detection of polymeric ECM substances [5]. Optotracing is based on a unique group of oligothiophene-based fluorescent tracer molecules that undergo a change in photophysical properties upon binding to repetitive motifs in macromolecules such as amyloid proteins and polysaccharides [5,29,30]. Binding to a macromolecular target induces a shift in the peak wavelength of absorption (Ex. *λ*_max_) and/or a change in fluorescence intensity (RFU). Using the first generation optotracers in our earlier *Salmonella* Enteritidis (*S.* Enteritidis) biofilm study, we observed a consistent red shift in Ex. *λ*_max_ and increased RFU when the optotracer bound to curli and cellulose [5]. By monitoring the kinetic appearance of ECM components in real-time, optotracing provided phenotypic evidence of the association of ECM assembly with stationary phase, corroborating transcriptomics studies showing increased expression of biofilm associated genes during this growth phase. Optotracing was also developed as a diagnostic application for biofilm infections via detection of native cellulose in urine from patients suffering from urinary tract infection [31]. Recently, a new generation of chemically modified optotracers, the EbbaBiolight series, has become commercially available, offering a selection of fluorescent tracer molecules with different peak emission wavelengths. One major improvement of such optotracers is the lack of fluorescence in the unbound state, making the optotracer largely invisible until a target appears and binding occurs. The combination of increased fluorescence intensity and spectral shifts upon binding allows for simultaneous detection of multiple macromolecules within a single sample [[32], [33], [34]].

Here, we explore the photophysical properties of an EbbaBiolight tracer molecule to develop a method for semi-high throughput detection and monitoring of biofilms growing at the air-solid interface on agar plates. By applying the optotracer to small-volume agar plates, this method converts the visual qualities of the biofilm morphotype into quantitative figures that can be analysed in real-time with minimal user bias. By applying the method on a biofilm model based on isogenic strains of *Salmonella* Enteritidis, we profile the spatial and temporal development of curli fibrils in surface-associated biofilm microcolonies and examine the formation of physically visible architectural elements of the *rdar* morphotype in relation to bacteria growth phases in the horizontal plane.

## 2 Materials and methods

### 2.1 Bacterial strains, media, and supplements

Bacterial strains and plasmids used in this study are shown in Supplementary Table 1. Strains include *S.* Enteritidis wild-type strain 3934 (wt) [35], the isogenic mutants Δ*csgD* [36], Δ*bcsA* [35], Δ*csgA* [37], and the clinical uropathogenic *Escherichia coli* strain UPEC No.12 [38]. When indicated, strains were transformed with the low-copy number plasmid p2777 [39,40], or pFPV25.1 [41], to enable stable GFP expression. Bacteria were routinely cultured on Luria-Bertani (LB) agar or in LB broth at 37 °C. For *Salmonella* biofilm assays, 1–25 μl of overnight cultures grown in LB broth without (w/o) salt were used to inoculate LB agar w/o salt, which then were incubated at 28 °C. When indicated, cultures growing exponentially at 37 °C under shaking conditions were used as inoculum. UPEC biofilm assays were performed at 37 °C. As indicated, media supplemented with Ampicillin (Amp, 100 μg/ml), Congo red (CR, 40 μg/ml) together with Coomassie brilliant blue G-250 (20 μg/ml; Sigma-Aldrich, Stockholm, Sweden), Live/Dead stain (0.5 μl/ml; ThermoFischer, Stockholm, Sweden), and EbbaBiolight 680 (Ebba680, 2 μl/ml) (Ebba Biotech, Stockholm, Sweden) were used. Ebba680 is an oligothiophene-based structural probe which upon binding to macromolecules emits light in the red and far-red spectrum. The molecular structure of optotracers in the EbbaBiolight series is proprietary to the supplier.

### 2.2 Expression of recombinant CsgA and preparation of CsgA fibrils

Using a pET11d expression vector, CsgA wt from *E. coli* was expressed in *E. coli* BL21(DE3) essentially as described [42]. The CsgA construct included the mature sequence (Met1 followed by N22 and the 5 imperfect repeats) but not the 20-residue signal sequence. To facilitate purification, a His_6_ sequence was added immediately after the C-terminal HQY sequence of CsgA. LB broth supplemented with Amp (LB-Amp) was inoculated with one colony of transformed cells. After 10 h incubation at 37 °C, bacteria were spread on LB-Amp agar plates and grown to confluency overnight. Cells were then transferred to LB-Amp medium and grown under shaking condition to OD_600_ = 0.8, when protein expression was induced with 1 mM IPTG. Following growth for 3–4 h, cells were harvested, the pellet was resuspended in lysis buffer (8 M GdmCl, 50 mM Tris-Cl pH 8.0), and the suspension was stirred overnight at 4 °C. Following centrifugation at 15,000 RPM, the supernatant was incubated with Ni-NTA agarose beads for 1.5 h at 4 °C. The beads were then packed in a PD-10 column (GE Healthcare, Brondby, Denmark), washed once with lysis buffer, followed by two consecutive washes in lysis buffer containing 15 mM and 30 mM imidazole, respectively. CsgA was then eluted with lysis buffer containing 500 mM imidazole.

Immediately before fibrillation, the CsgA buffer was exchanged into phosphate buffered saline (PBS) pH 7.4 using PD-10 desalting columns. Protein concentration was estimated using OD_280_ and an extinction coefficient of 11,460 M^−1^ cm^−1^ (*i.e.* 1 mg/ml has an OD_280_ of 0.824). CsgA was incubated at 0.5 mg/ml in an Eppendorf shaker at 37 °C at 300 RPM for 48 h (sufficient to induce complete fibrillation [17]), pelleted and resuspended to 1 mg/ml in Milli-Q water.

### 2.3 Congo red biofilm assay, calcofluor assay, and live/dead assay

CR-containing agar (CR-agar) was prepared by supplementing LB agar w/o salt with CR and Coomassie brilliant blue G-250. When indicated, CR-agar was supplemented with Ebba680 (2 μl/ml). A volume of 20 ml CR-agar was used in Petri dishes, while 2 ml was used per well in sterile, Costar tissue culture treated 6-well plates (Sigma-Aldrich, Stockholm, Sweden). Biofilm formation was initiated by positioning 10 μl aliquots from liquid, stationary phase cultures onto CR-agar in the Petri dish and in wells of the 6-well plate. After incubation at 28 °C for 3–4 days, morphotypes based on curli and cellulose expression were determined by visual inspection and documented by photography. To perform the Calcofluor assay, LB agar w/o salt supplemented with 2 μl Calcofluor White stain (Calcofluor White M2R (1 g/L), Evans blue (0.5 g/L), Sigma-Aldrich, Stockholm, Sweden) per ml agar was used in the 6-well plate format. For fluorescence-based identification of live and dead bacteria in the biofilms, LB agar w/o salt was supplemented with the Live/Dead stain using 1 μl each of SYTO®9 and propidium iodide (PI) per ml agar in the 6-well plate format. Biofilms in the Calcofluor assay and Live/Dead assay were allowed to form according to the same procedure as used in the CR assay and analysed by automated microscopy (see below).

### 2.4 Optotracer-based 6-well plate biofilm and curli assays

**Fig. 1:**
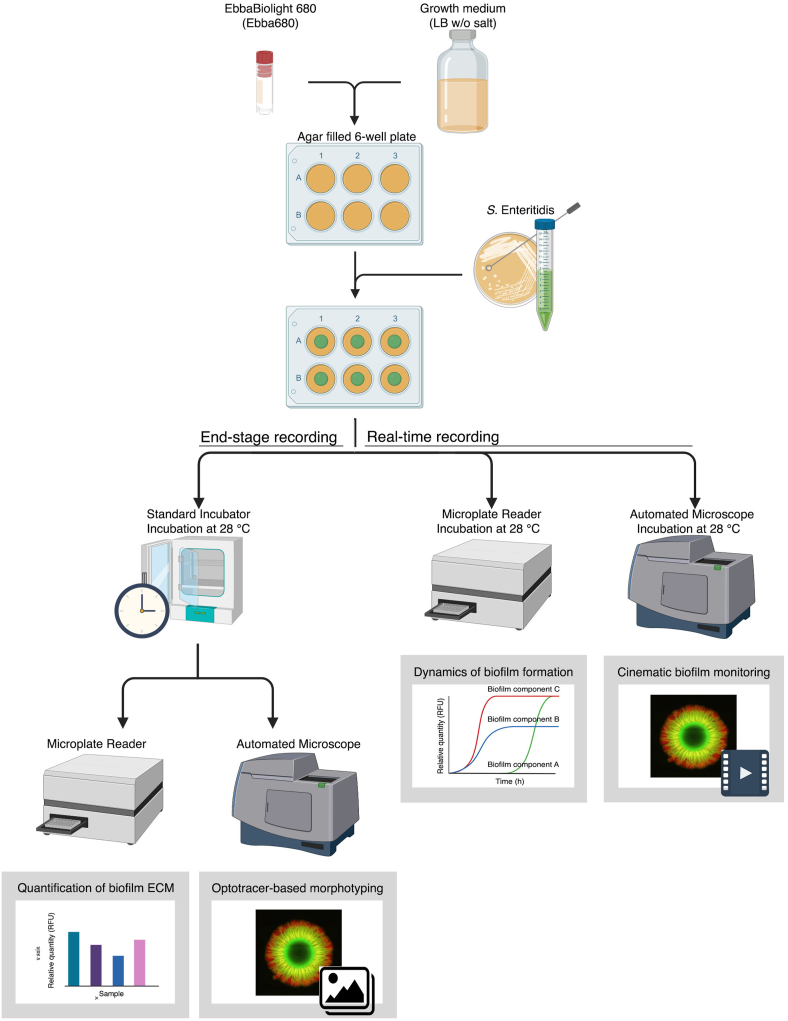
**Workflow of the 6 well-plate optotracing biofilm method.** Wells are filled with 2 ml LB agar without salt, supplemented with EbbaBiolight 680. The bacterial inoculum (1–25 μl) is placed on the agar centre. The plate is incubated in different instruments depending on experiment. ***End-stage recording:*** Following incubation within a standard incubator, formed macrocolonies and biofilms are analysed in a microplate reader or an automated microscope. ***Real-time recording:*** Dynamics of biofilm formation is studied by incubating the plate in a temperature-controlled microplate reader, programmed to record spectra at defined time intervals. Alternatively, the plate is incubated in a temperature-controlled automated microscope that provides a cinematic view of biofilm formation over ca 70 h.

**Fig. 2:**
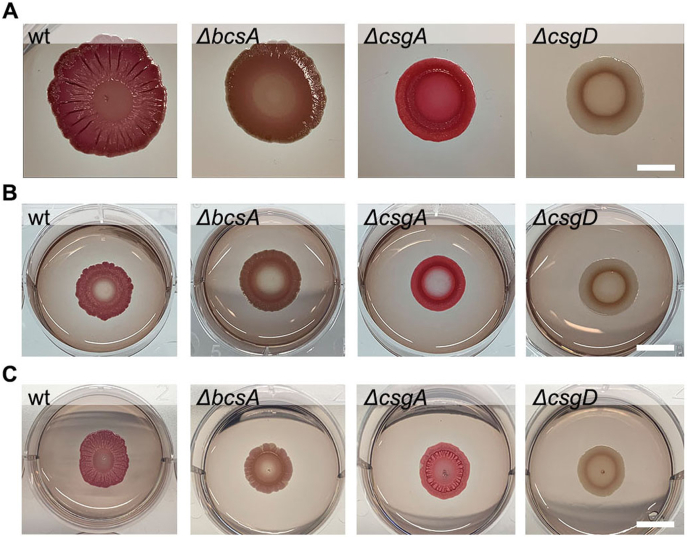
**Colony morphotypes on Congo red assays.** Photographs of biofilm colonies of wt, *ΔbcsA*, *ΔcsgA*, and *ΔcsgD* in **(A)** Petri-dish CR-agar assays, **(B)** 6-well plate CR-agar assays, and (**C**) 6-well plate assays using CR-agar supplemented with EbbaBiolight 680. Scale bar = 10 mm. (For interpretation of the references to colour in this figure legend, the reader is referred to the Web version of this article.)

As depicted in Fig. 1, Fig. 2 ml of LB agar w/o salt supplemented with the fluorescent tracer molecule Ebba680 was added per well in sterile Costar tissue culture treated 6-well plates (Sigma-Aldrich, Stockholm, Sweden). Biofilm formation was initiated using 10 μl inoculum from stationary phase cultures, positioned centrally on the agar. When assessing potential effects of inoculum volume on ECM formation, inoculums of 1, 5, 10, 15, 20 and 25 μl were used. Plates were incubated at 28 °C for 3–4 days. Depending on experiment, incubation was performed in an ordinary incubator, spectrophotometer, or automated microscope. When evaluating if Ebba680 binds curli, 10 μl purified curli fibrils (1 mg/ml) was positioned centrally on the agar in wells of the 6-well plate and analysed by automated microscopy.

### 2.5 Automated microscopy

Fluorescence imaging of biofilms (cells and ECM) and purified curli in the 6-well plate was achieved by microscopy in a Lionheart™ FX Automated Microscope (Ramcon, Sweden), using the software Gen5 (Ramcon, Sweden). To detect calcofluor fluorescence, the microscope was loaded with a 1.25× objective, Plan Apochromat WD 5 NA 0.04, and 365 nm LED cube/DAPI filter cube. To detect bacterially expressed GFP or SYTO®9, the microscope was loaded with a 1.25× objective, Plan Apochromat WD 5 NA 0.04, and 465 nm LED cube/GFP filter cube. To detect Ebba680 fluorescence, the microscope was loaded with a 1.25× objective and a 523 nm LED cube/propidium iodide filter cube. The LED intensity, integration time and gain were 5; 60; 12 for GFP detection, and 5; 600; 10 for Ebba680 detection. Brightfield images were recorded using software pre-settings. Images were acquired under ‘Tiling’ mode, wherein optical signals from the entire well is collected as a 5-by-4 grid that is subsequently ‘stitched’ together during post imaging analysis. As reference signal for auto-focusing and ‘stitching’, we used either GFP fluorescence from the biofilm, or Ebba680 fluorescence. During real-time monitoring of biofilm growth, the Lionheart™ FX Automated Microscope was programmed with additional settings for temperature control and kinetic imaging, to maintain a constant 28 °C environment and automatic imaging for up to 70 h at 2 h intervals.

### 2.6 Confocal laser scanning microscopy of biofilm regions

Biofilms formed by wt-p2777 and Δ*csgD*-p2777 in the 6-well plate biofilm assay containing Ebba680 were gently sampled using a 10 μl sterile loop (VWR, Stockholm, Sweden) and placed on a glass slide (VWR, Sweden). One drop of DAKO mounting medium (ThermoFisher, Stockholm, Sweden) was added and the sample was sealed with a glass coverslip (VWR, Stockholm, Sweden) prior to analysis by fluorescence confocal microscopy (FV1000 Confocal Microscope, Olympus, Sweden) using a UPLSAPO 63 × W (NA 1.2) water immersion lens (Olympus, Sweden). To detect bacteria-expressed GFP and ECM-bound Ebba680, factory pre-settings of filters for GFP and propidium iodide were applied. Sequential acquisition in ‘Frame’ mode was selected during imaging. Phase contrast images were collected in the transmitted light detector with excitation at 473 nm.

### 2.7 Live biofilm monitoring by spectrophotometry

Fluorescence recording of ECM-bound Ebba680 was achieved by spectrophotometry using a Synergy Mx Monochromator-Based Multi-Mode Microplate Reader (Biotek, Bad Friedrichshall, Germany). Temperature control was set to maintain a constant 28 °C environment. Bacteria culture density was monitored by GFP fluorescence (Ex. λ 445 nm, Em. λ 510 nm) for a period of 72 h at 15 min intervals, via a 15 × 15 area scan (circle-filled) performed at each time point. Simultaneously, Ebba680 fluorescence (Ex. λ 535 nm, Em. λ 660 nm) was recorded. Time series were obtained by selecting the 4 areas H8 – H11 from the 15 × 15 grid spanning from the centre of the inoculum applied at 0 h, to the edge of the biofilm at 72 h. Readings for GFP and Ebba680 at each point were presented in the same graphs to visualize the hourly change in signals.

### 2.8 Statistical analysis

Data was organized and processed with GraphPad Prism 8 (Graphpad Software, La Jolla, CA, USA).

### 2.9 Preparation and analysis of images

Fiji (Wisconsin, USA) [43] was used to process and perform orthogonal analysis of image stacks acquired by confocal microscopy, and to analyse the physical dimensions of biofilms in images collected by automated microscopy.

## 3 Results

### 3.1 Detection of the ECM architecture by an optotracer assay

To enable unbiased, semi-high throughput analysis of biofilm development over a period of several days, we had to develop a multi-well format biofilm assay compatible with automated microscopy. For this purpose, we used the prevailing biofilm assay, the Congo red assay, as a foundation for improvement. We therefore transformed the standard Petri-dish format, usually containing 20 ml Congo red (CR)-supplemented LB agar w/o salt, into a small-volume, 6-well plate format. With 2 ml CR-agar added to each well, we studied if the reduced size of the wells had any influence on the development of biofilm morphotypes formed by an isogenic collection of *S.* Enteritidis strains. The set of strains included the wild-type (wt) strain 3934 (curli+, cellulose+), the Δ*bcsA* (curli+, cellulose-) and Δ*csgA* (curli-, cellulose+) mutants, as well as the Δ*csgD* mutant (curli-, cellulose-) (Supplementary Table 1). Depending on curli and/or cellulose expression, this strain collection is known to present typical biofilm morphologies based on the colour, roughness, and dryness of biofilms formed on CR-agar [44]. *Salmonella* biofilms are accordingly often categorized into the morphotypes *rdar* (red, dry and rough) for strains expressing curli fimbriae and cellulose, *bdar* (brown, dry and rough) for strains expressing curli fimbriae, and *pdar* (pink, dry and rough) for strains expressing cellulose [44,45]. From overnight cultures, we inoculated 10 μl of each strain onto the CR-agar in individual wells of the 6-well-plate, and on the archetypical Petri-dish. After 4 days of incubation at 28 °C, we photographed each biofilm. Irrespective of assay format, biofilms formed by wt (curli+, cellulose+), Δ*bcsA* (curli+, cellulose-) and Δ*csgA* (curli-, cellulose+) developed the *rdar, bdar*, and *pdar* morphotypes as expected from their respective genotypes, while Δ*csgD* (curli-, cellulose-) consistently presented a white, supple and smooth morphology (Fig. 2A and B). The cellulose expression pattern of the strains was also confirmed by the Calcofluor assay (Supplementary Figs. 1A–D). Thus, reduction in agar volume did not negatively affect nor alter the biofilm morphotypes. We observed, however, that biofilms in the Petri-dish assay differed in size, with the wt forming larger biofilm than those formed by Δ*bcsA*, Δ*csgA* and Δ*csgD* mutants. This indicates that curli and cellulose expression may influence the ability to spread on the agar surface. Biofilms formed in the 6-well plate assay were all round and of similar size. This uniformity allowed for easy assessment of biofilm morphologies, which is likely to benefit high throughput analysis of biofilms and biofilm development.

**Fig. 3:**
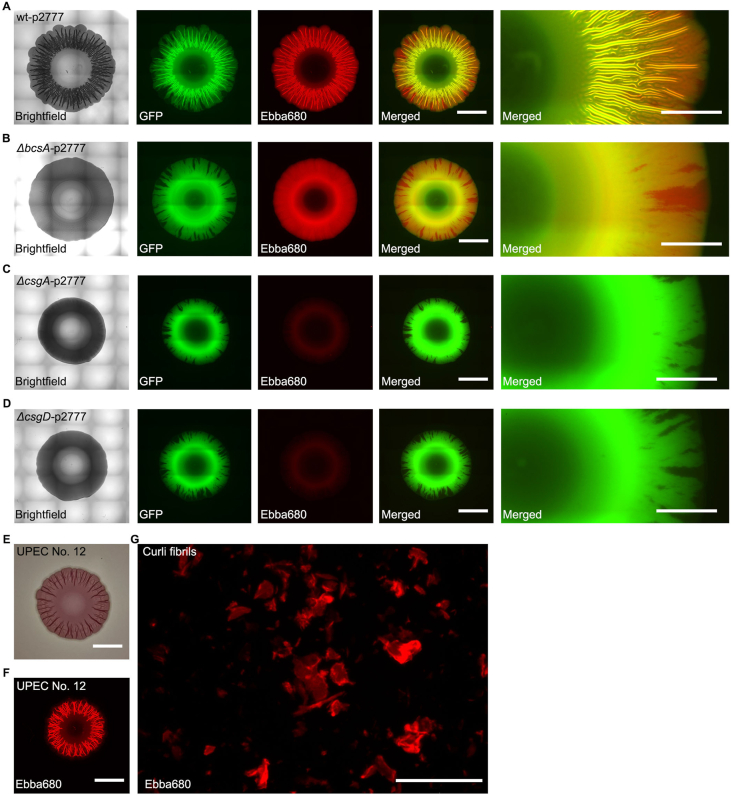
**Visualization of air-solid biofilms in 6-well agar plates**. Automated microscopy showing biofilms formed using the 6-well plate optotracing method, in which bacteria grow on agar supplemented with Ebba680. Morphologies of the GFP-expressing strains **(A)** wt-p2777 (curli+, cellulose +), **(B)***ΔbcsA*-p2777 (curli+, cellulose-), **(C)***ΔcsgA*-p2777 (curli-, cellulose+), and **(D)***ΔcsgD*-p2777 (curli-, cellulose-) are shown in brightfield images, while fluorescence microscopy shows the spatial distribution of bacteria (GFP, green) and ECM curli (Ebba680, red) separately and in merged images of respective strain. Scale bar = 5 mm. Enlarged sections from the merged images of the respective strain are shown to the right. Scale bar = 2 mm. **(E)** Photograph showing the colony morphotype of uropathogenic *E. coli* strain UPEC No.12 in a 6-well plate CR-agar assay. Scale bar = 5 mm. **(F)** Automated fluorescence microscopy of UPEC No.12 using the 6-well plate optotracing method shows the spatial distribution of ECM curli (Ebba680, red) in the biofilm. Scale bar = 5 mm. **(G)** Automated fluorescence microscopy showing purified curli fibrils (Ebba680, red) manually pipetted directly onto Ebba680-supplemented agar in the 6-well plate assay. Scale bar = 200 μm. (For interpretation of the references to colour in this figure legend, the reader is referred to the Web version of this article.)

While earlier optotracers enabled real-time tracing of *Salmonella* biofilm in liquid cultures based on the spectral shifts optotracers undergo when they bind to the ECM [5], current optotracers show inducible fluorescence. The fluorescent signal from these optotracers remains off until the biomolecular binding target appears, allowing binding to occur. We took advantage of this photophysical feature unique to optotracers when investigating if the lack of fluorescence of unbound optotracers would allow us to establish a biofilm assay on solid medium, in which fluorescent signals only are emitted from optotracers binding to the ECM as it forms. To enable fluorescence-based imaging of bacteria and the ECM, we used the optotracer Ebba680 which emits light in the red to far-red range and is therefore compatible with GFP. This led us to expect that GFP-expressing bacterial cells and Ebba680-stained ECM macromolecules could be simultaneously visualized. Accordingly, we set up a workflow in which we prepared 6-well plates containing 2 ml LB agar w/o salt supplemented with the optotracer Ebba680 (Fig. 1). To enable fluorescent co-detection of bacterial cells and ECM, we used the aforementioned collection of *S.* Enteritidis strains, modified with a low-copy number vector to allow for constitutive expression of GFP (wt-p2777, Δ*bcsA*-p2777, Δ*csgA*-p2777, Δ*csgD*-p2777) (Supplementary Table 1). We inoculated 10 μl from overnight cultures of each strain onto the agar of 6-well plates, which were incubated for 4 days at 28 °C. Plates were then placed in an automated microscope for end-stage analysis of the formed biofilms. In accordance to an experiment showing that the *rdar* morphotypes develop irrespective of the presence of Ebba680 in the agar, (Fig. 2C), the brightfield image of wt-p2777 showed a morphotype similar to that grown on the CR-agar plates (Fig. 3A). Fluorescence imaging in the GFP channel revealed distinct fluorescence from GFP-expressing bacteria, varying in intensity throughout the biofilm (Fig. 3A, GFP). Relative to the entire biofilm, the central core harbouring the oldest cells showed low fluorescence. This region was bordered by a ring of intense fluorescence, which we refer to as the intermediate region. The strong signal gradually diminished in the intermediate region towards the fringe at the periphery of the biofilm. A lack of GFP expression was sporadically observed at the fringe. To test if this lack was due to loss of the β-lactamase encoding plasmid p2777, we repeated the experiment on selective, Amp-containing media. A similar pattern was observed irrespective of the antibiotic (Supplementary Fig. 2), suggesting that the lack of GFP expression results from spontaneous mutations within the plasmid, which persists in subsequent generations as reported previously [46]. By applying standard filters on the microscope, red fluorescence from Ebba680 binding to the ECM matrix was readily observed (Fig. 3A, Ebba680). In contrast, no signal was seen from agar outside the bacterial colony. This illustrates the unique feature of the new generation optotracers, which becomes fluorescent only when target macromolecules appear to which the optotracer binds. The red staining of ECM showed distally projecting radial patterns. Ridges commonly observed in CR morphotyping were visible as streaks running perpendicular to the radial patterns. Co-localization of GFP and Ebba680 fluorescence revealed wall-like structures composed of cells and ECM organized into a network of channels (Fig. 3A, Merged). Detailed analysis at higher magnification showed weaker Ebba680 signal in the channel lumens, indicating a comparatively lower amount of ECM despite stronger GFP presence.

Biofilms formed by Δ*bcsA*-p2777 (curli+, cellulose-) showed an expected morphotype in the brightfield image (Fig. 3B). Fluorescence microscopy revealed that bacteria had formed a radial ECM pattern, with the intermediate region exhibiting the highest Ebba680 fluorescence signal (Fig. 3B, Ebba680). Co-localization of GFP and Ebba680 fluorescence at low and high magnification revealed, however, that this strain is unable to form channel-like structures (Fig. 3B, Merged). Yet a different picture appeared when analysing biofilms grown from Δ*csgA*-p2777 (curli-, cellulose+) and Δ*csgD*-p2777 (curli-, cellulose-). These biofilms did not show any notable Ebba680 fluorescence when studied individually or in merged images (Fig. 3C and D). Since both strains are genetically incapable of curli expression, the data implies that the one remaining binding target for Ebba680 in the current comparison of these isogenic mutants is the curli fibril. *E. coli,* like *Salmonella*, express curli amyloid fibers as a major ECM component. To determine if Ebba680 would also bind *E. coli* ECM-curli, overnight cultures of the clinical uropathogenic *E. coli* strain UPEC No.12 was inoculated onto CR-agar and Ebba680-containing agar in the 6-well plate format. Brightfield images showed that UPEC No.12 developed the *rdar* morphotype (curli+, cellulose+) on CR-agar after 2 days incubation, indicating the expression of curli and cellulose in the biofilm colony (Fig. 3E). When grown on Ebba680-containing agar, fluorescence microscopy revealed red staining of the ECM throughout the biofilm colony, in a radial pattern identical to Ebba680 stained *Salmonella* wt-p2777 biofilm colonies (Fig. 3F). This suggests that Ebba680 was equally sensitive to *E. coli* amyloid fibers. To further confirm that Ebba680 is able to bind curli, we added purified curli fibrils onto Ebba680-containing agar in the 6-well plate format. Automated imaging using the same optical settings as above showed intense red fluorescence, confirming efficient Ebba680-staining of the purified curli fibrils (Fig. 3E).

### 3.2 Non-uniform location of curli fibrils in the biofilm

**Fig. 4:**
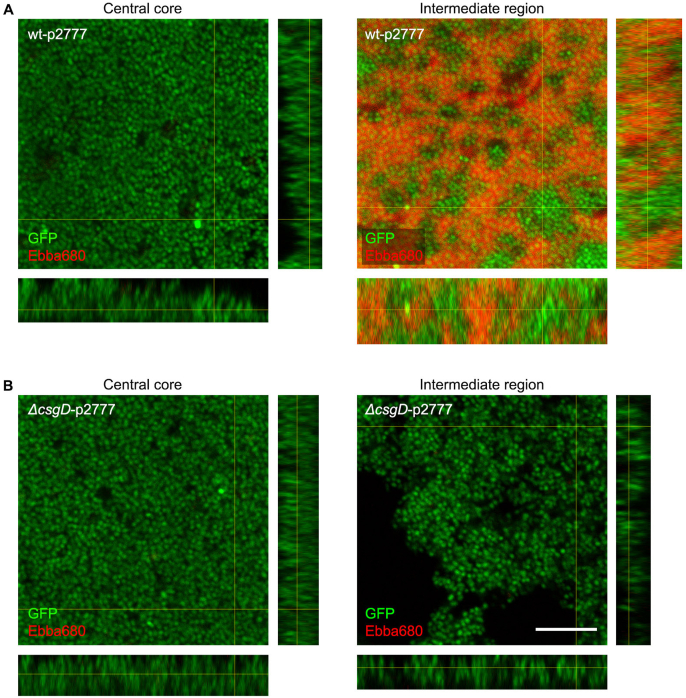
**Curli organisation in different regions of the biofilms**. Merged projections of image stacks collected by confocal fluorescence microscopy, showing the spatial distribution of bacterial cells (GFP, green) and curli (Ebba680, red) in **(A)** the central core (10.6 μm stack, z-step = 0.53 μm) and intermediate region (21.2 μm stack, z-step = 0.53 μm) of biofilm formed by wt-p2777 (curli+, cellulose+), and **(B)** the central core (5.3 μm stack, z-step = 0.53 μm) and intermediate region (6.36 μm stack, z-step = 0.53 μm) of biofilm formed by *ΔcsgD*-p2777 (curli-, cellulose-). Side projections are shown for each image stack. Scale bar = 10 μm. (For interpretation of the references to colour in this figure legend, the reader is referred to the Web version of this article.)

In the intermediate region, automated microscopy showed distinct radial organisation of the ECM, a structure absent in the central core. To investigate this, we transferred samples from the central core and the intermediate regions of biofilms formed by wt-p2777 (curli+, cellulose+) onto microscope slides and performed confocal microscopy. Since the *rdar* morphotype of *Salmonella* biofilms depends on both curli and cellulose expression, we used Δ*csgD*-p2777 (curli-, cellulose-) for comparison from here and onwards, as this strain lack both these ECM components. Since the optotracer had bound to the biofilm as it formed on the Ebba680-supplemented agar, fluorescence imaging could be directly performed without additional stains. The workflow of this experiment is depicted as “End-stage recording” in Fig. 1. Samples were analysed for bacterial cells (GFP) and curli fibrils (Ebba680). In biofilm formed by wt-p2777, the central core showed a continuous layer of GFP expressing bacteria, with no visible indications of curli production (Fig. 4A). The green fluorescence was consistent throughout the full thickness of the biofilm, as observed in side projections of the image stack. The intermediate region showed a distinctly different pattern. Intense red fluorescence showed GFP-expressing bacteria surrounded by a dense matrix of curli-based ECM. The red fluorescence was, however, not uniformly distributed. Multiple areas were void of the red signal, implying an absence of curli-based ECM. This patchy ECM distribution was maintained throughout the 3D structure of the biofilm, as shown by the orthogonal views of the image stack, suggesting that curli assembly only occurs on a subpopulation of cells in the biofilm. In biofilm formed by the mutant Δ*csgD*-p2777, dense aggregation of bacterial cells was observed in the central core and the intermediate region (Fig. 4B). Red fluorescence was not observed in any of the regions, confirming the inability of this strain to produce the ECM components curli and cellulose.

### 3.3 Physical dimensions influence the ECM architecture

**Fig. 5:**
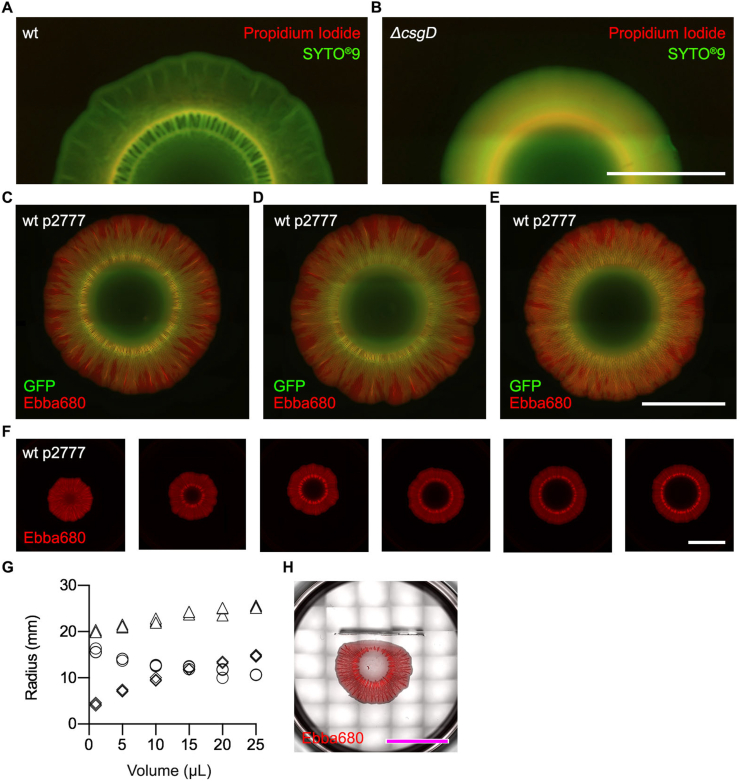
**ECM organization is influenced by surface availability but not the inoculum size**. Automated fluorescence microscopy shows live and dead cells in biofilms formed by **(A)** wt and **(B)***ΔcsgD* (curli-, cellulose-) for 3–4 days in 6-well plates on agar supplemented with the Live/Dead stains SYTO®9 (green) and propidium iodide (red). Scale bar = 10 mm. **(C–F)** Automated fluorescence microscopy of biofilms formed in the 6-well plate optotracing assay, in which bacteria grow on agar supplemented with Ebba680. Merged images show the spatial distribution of curli (red) and bacteria (green) in biofilm formed by wt-p2777 using **(C)** overnight culture, **(D)** centrifuged, resuspended overnight culture, and **(E)** exponential phase culture as inoculum. Scale bar = 10 mm. **(F)** Effects of inoculum volumes, varying between 1 and 25 μl, of strain wt-p2777 on the spatial distribution of curli ECM (red) in biofilms growing in 6-well agar plates supplemented with Ebba680 (scale bar = 10 mm). **(G)** The radius (mm) of the centre region (diamond), the intermediate region (circle) and the full size (triangle) of the biofilm macrocolony. **(H)** The effect of area restriction on biofilm development. Area restriction is introduced by vertical insertion of a sterile glass coverslip into the agar nearby the inoculum site. The merged image shows the wt-p2777 biofilm macrocolony (phase contrast) and the spatial distribution of curli (red in fluorescence image) after 4 days incubation in the presence of the obstacle. Scale bar = 10 mm. (For interpretation of the references to colour in this figure legend, the reader is referred to the Web version of this article.)

A biofilm is composed of live and dead bacterial cells. To analyse whether the radial organisation of ECM-rich and -poor regions associates to the location of dead bacteria in the biofilm, we used the “End-stage recording” workflow depicted in Fig. 1. We prepared a 6-well plate assay containing LB agar w/o salt supplemented with the Live/Dead stains SYTO®9 and PI. To avoid interference with green fluorescence from GFP, we used the wt and Δ*csgD* strains without GFP-expression as we inoculated 10 μl overnight cultures onto the agar. The 6-well plate was incubated for 3–4 days at 28 °C, then imaged by automated microscopy. The wt biofilm showed most intense green (SYTO®9) and red (PI) fluorescence in the intermediate region, reflecting a high density of biomass (Fig. 5A). Green fluorescence was clearly distributed throughout the biofilm, indicating the presence of live cells in the entire biofilm. In contrast, no obvious locations with accumulated orange fluorescence were detected, suggesting that dead cells do not accumulate in any specific region. This implies that the radial organization of curli fibrils in wt biofilm forms independently from the location of dead cells. A different picture emerged from Live/Dead analysis of the Δ*csgD* mutant (curli-, cellulose-) (Fig. 5B). This biofilm showed green fluorescence in the central core region, bordered by alternating rings of orange and green fluorescence that repeated until the fringe at the periphery. This indicated a radial organization of regions with high and low density of dead cells. By comparing the patterns from wt and Δ*csgD* biofilms, it appeared that the lack of ECM curli and cellulose impacts cell viability, evident from the accumulation of regions rich in dead cells.

To determine whether the formation of radial structures of the biofilm is influenced by the growth phase of the starting bacteria, we used 10 μl inoculums harvested from exponential (OD_600_ = 0.5) and stationary phase wt-p2777 cultures. Also, we tested whether metabolites accumulated during pre-inoculum growth might influence biofilm development by washing stationary phase cells prior to their addition onto the agar. The inoculated 6-well plates containing LB agar w/o salt supplemented with Ebba680, were incubated at 28 °C for 4 days. Automated microscopy showed the typical radial organisation of the ECM structures in the biofilms irrespective of inoculum (Fig. 5C–E). Likewise, the total surface area and the radius remained the same for the central core and the intermediate region in the three conditions. This suggested that biofilm morphologies develop independently of the inoculums’ growth phase and that ECM development is not hindered by metabolites from the stationary phase cultures. We then analysed if the volume of inoculum influenced the biofilm morphology. We applied 1, 5, 10, 15, 20, and 25 μl of overnight cultures of wt-p2777 onto the Ebba680-based 6-well plate assay and allowed biofilms to develop for 4 days at 28 °C. Automated microscopy showed progressively larger central cores as the volume of the inoculum increased (Fig. 5F). We quantified this using the ImageJ software, as we measured the radius of the central core, the intermediate region, and the total biofilm macrocolony at each inoculum size. We detected progressively larger radii of the central cores relative to the total biofilm radii as the volume of the inoculum increased (Fig. 5G). In contrast, the radii of the intermediate regions decreased as inoculum volumes increased. Despite an inverse relationship between inoculum volume and the quantity of curli ECM, our observation suggests that bacteria with the capacity to form biofilms would likely do so within this assay. To study if this pattern reflects the limited area on which biofilm can form within the wells, we inserted a physical obstruction, *i.e.* a sterile glass coverslip, into the agar prior to inoculation. Analysis after 4 days showed a non-uniform pattern, as the circularity of the biofilm macrocolony was obviously abrogated (Fig. 5H). The intermediate region in the side facing the obstruction was less wide compared to the opposite non-obstructed side, while the central core was unaffected. Importantly, the fringe at the periphery of the biofilm never contacted the glass coverslip despite close proximity. This suggests that in the present model, bacteria growing in the biofilm lifestyle are able to recognise the physical dimensions, avoiding obstructions while maximizing area coverage.

### 3.4 Formation dynamics of the ECM architecture viewed in the horizontal plane

**Fig. 6:**
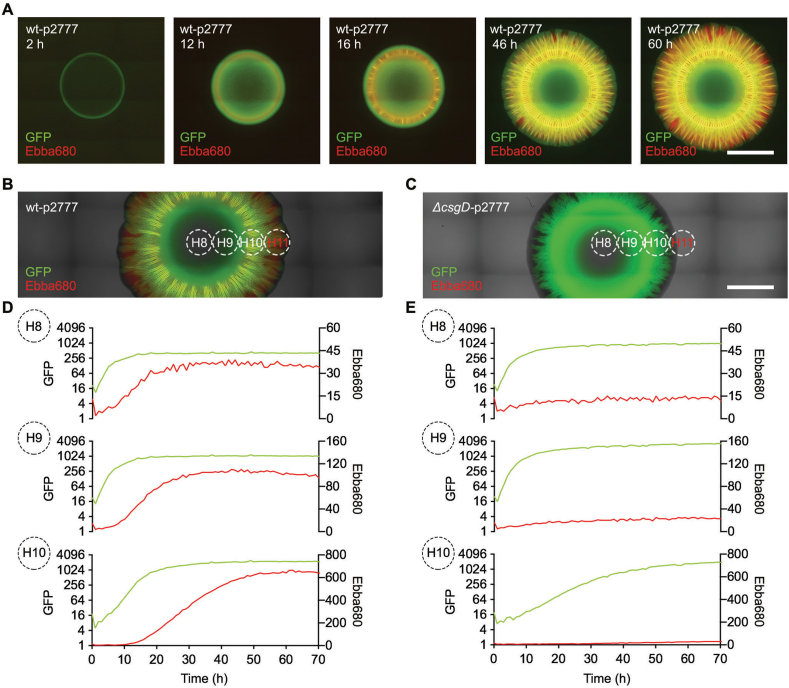
**Kinetics of biofilm growth and ECM expression at the air-solid interface**. Automated fluorescence microscopy of biofilms formed in 6-well agar plates supplemented with Ebba680. **(A)** The spatial distribution of bacterial cells (GFP, green) and curli ECM (Ebba680, red) of strain wt-p2777 growing for 60 h in the automated microscope, with images collected at 2 h intervals. Representative fluorescence micrographs at 2, 12, 16, 46 and 60 h are shown. An animation of all images showing real-time growth of the biofilm is found in Supplementary Video 1. Scale bar = 5 mm **(B–C)** Fluorescence micrographs acquired by automated microscopy showing **(B)** wt-p2777 (curli+, cellulose+) and **(C)***ΔcsgD*-p2777 (curli-, cellulose-) biofilm macrocolonies at the end of 72 h spectrophotometric live recording of biofilm formation. Circled areas show estimated positions of regions (H8 = inner central core; H9 = outer central core; H10 = intermediate region) selected for kinetic analysis by spectrophotometric recordings. H11 at the fringe of the periphery was recorded but not analysed, therefore indicated in red. Scale bar = 5 mm. **(D**–**E)** Spectrophotometric real-time recordings of macrocolony biofilm formation of **(D)** wt-p2777 (curli+, cellulose+) and **(E)***ΔcsgD*-p2777 (curli-, cellulose-). Data were acquired every 15 min for 70 h during incubation at 28 °C. GFP fluorescence (Ex. λ 445 nm, Em. λ 510 nm), plotted on a logarithmic scale, shows bacterial growth (green line, left y-axis), and Ebba680 fluorescence (Ex. λ 535 nm, Em. λ 660 nm) reports curli ECM production (red line, right y-axis). Graphs named H8 – H10 show recordings specific for individual regions of the biofilm macrocolonies indicated in panel **(B, C)**. Graphs are representative of 3 experimental repeats composed of 2–3 technical replicates. (For interpretation of the references to colour in this figure legend, the reader is referred to the Web version of this article.)

Since the presented assay is compatible with automated microscopy, the dynamics of ECM formation can be studied in real-time. To study the spatial-temporal relationship between bacterial growth phases and ECM formation, we used the “Real-time recording“ workflow presented in Fig. 1. We prepared a 6-well plate with Ebba680-supplemented agar and inoculated each well with wt-p2777. Plates were incubated inside the automated microscope at 28 °C, allowing brightfield and fluorescence images to be collected every 2 h for a total period of 60 h. Visualising bacterial growth based on GFP-expression and curli fibrils by Ebba680 fluorescence, the outward expansion of the biofilm was readily observed (Fig. 6A, Supplementary Video 1). Green fluorescence at the periphery of the inoculum, indicating significant growth of the bacterial colony, became visible after a lag time of circa 2 h. Over the next 10 h, the green fluorescence intensified and formed the typical ring-like pattern. The appearance of red fluorescence at 12 h suggested that bacteria now had started to produce curli fibrils, which intensified throughout the growth of the biofilm. The first channel-like structures of the intermediate region became visible at 16 h. These channels gradually lengthened as the biofilm surface area continued to expand. After an additional 30 h, the intermediate region appeared to be fully formed, and no visible changes in the overall size of the biofilm were later observed. Throughout the full 60 h length of this experiment, the fringe at the periphery of the biofilm always predominantly fluoresced in green, indicating that growth of metabolically active, GFP-producing bacteria always precedes expression of ECM.

Supplementary video related to this article can be found at https://doi.org/10.1016/j.bioflm.2021.100060

The following is/are the supplementary data related to this article:

Video 1 Video 1

In an attempt to define the growth characteristics of a biofilm in the horizontal plane, we simultaneously analysed the time courses of bacterial growth and ECM formation. In accordance to the workflow “Real-time recording“ (Fig. 1), we inoculated 6-well plates containing Ebba680-supplemented LB agar w/o salt with wt-p2777 and Δ*csgD*-p2777, respectively, and placed the plate inside a 28 °C multi-mode microplate reader. Over the next 72 h, we performed spectrophotometric recordings every 15 min of the entire area of each well by applying a 15 × 15 grid pattern to the fluorescence scan (Supplementary Fig. 3). The GFP signal (bacterial growth) was obtained using excitation at 445 nm (Ex. λ) and emission at 510 nm (Em. λ), the Ebba680 signal (curli fibril expression) using Ex. λ 530 nm and Em. λ 660 nm. After 72 h, the 6-well plate was moved from the plate reader to the automated microscope in which images were obtained in order to identify grid coordinates representing the inner and outer parts of the central core, and the intermediate regions of the biofilms (Fig. 6B and C).

The central core of the inoculum corresponded to grid H8. By plotting the spectrophotometrically recorded fluorescence signals in H8 over time, wt-p2777 showed an exponential increase in GFP fluorescence for circa 10 h, which then entered a plateau (Fig. 6D). This indicated that inoculated cells rapidly enter a logarithmic growth phase without any apparent lag time. The appearance of curli, reported by Ebba680 fluorescence, coincided with the shift from late logarithmic to early stationary phase at circa 10 h. Curli production ceased during the following 10 h, eventually reaching a plateau that remained throughout the full length of the experiment. Grid H9 corresponded to the outer part of the central core. This area showed similar temporal patterns of bacterial growth and curli production as the centre of the inoculum. The fluorescence intensities of GFP and Ebba680 were, however, markedly increased, suggesting a possible higher biofilm density. The intermediate region corresponded to grid H10. As expected, the fluorescence intensity of this area was initially very low since no bacteria were present at this location at the start of the experiment. After circa 7 h, green fluorescence increased and transitioned into a pattern indicative of logarithmic growth, before entering a stationary phase circa 8 h later. Curli production, which was detected at the transition from late logarithmic to early stationary phase of GFP expression, persisted for approximately 30 h before entering a plateau. The long duration is justified by our observation of the abundant ECM structures in the intermediate region of the biofilm. Grid H11, representing the fringe at the periphery, was recorded but not presented due to the random loss of GFP expression at the edges of the macrocolony. Analysis of the mutant strain Δ*csgD*-p2777 (curli-, cellulose-) showed a similarly staggered GFP expression pattern in grids H8 – H10 as observed for wt-p2777, reflecting similar spreading of bacterial growth across the agar surfaces (Fig. 6E). However, no Ebba680 fluorescence was detected in any of the grid areas, demonstrating the inability of this mutant to produce curli and cellulose.

Taken together, the sigmoidal profile of GFP expression in biofilms formed by the wt and mutant strains indicates that the growing macrocolonies on solid media, viewed from a horizontal plane, show similar growth phases as what is observed in liquid cultures. Furthermore, our observation that ECM production always commenced after the late exponential phase of bacterial growth, irrespective of the location of the biofilm, suggests that ECM structures form on site after bacteria have colonized the surface.

## 4 Discussion

We demonstrate an optotracer-based 6-well agar plate biofilm assay as a semi-high throughput method for studies of biofilm formation at the air-solid interface. Central to this method is the fluorescent tracer molecule added to the LB agar, whose inducible fluorescent signal remains off until the biomolecular binding target appears. Binding of the optotracer to the ECM of the expanding biofilm therefore presents an on-like switching of fluorescence, readily detectable by microscopy and spectroscopy. This switch enables real-time recordings of ECM production, which primarily targets curli in *Salmonella* and *E. coli* used in the current model. Combined with GFP-expressing bacteria, the optotracer-based method enables mapping of the spatial-temporal relationship between ECM, its architecture from a horizontal plane, and the growth phases of bacterial cells in the multidimensional biofilm community.

We designed our semi-high throughput biofilm assay to improve on several aspects of current methods in biofilm research. Miniaturization of the Petri-dish assay to a 6-well plate format reduces the agar volume by circa 90%, leading to reduced consumption of media and supplements. The thin agar is compatible with microscopy, allowing the kinetics of biofilm growth on the agar to be visualized in a temperature-controlled automated microscope. This opens for automated imaging and image analysis to eliminate the main bottleneck of manual assessment of colony morphotypes in current methods. This is further helped by the small colonizable surface (85% less than in a Petri-dish), which promotes the development of uniformly sized biofilm colonies that are easy to compare and evaluate. Differential diffusion of the tracer molecule has not been noted. The small format may, however, limit the duration of the experiment due to the risk of desiccation. While this was not observed in our 4-day studies, it may become relevant at longer incubation times. We conclude that sufficient morphotypical features developed to allow clear assessments of bacterial cells and ECM in our *Salmonella* model, however this may vary with other species. The binary results generated by the inducible ECM fluorescence can potentially facilitate the development of fully automated biofilm analytics. A technology shift towards a fully automated workflow for biofilm analysis will likely benefit the biofilm research community and clinical microbiology laboratories alike.

The transition from planktonic to sessile cells is a complex, highly regulated process [47]. While many methods allow studies of bacterial physiology in liquid cultures, few can be applied to monitor biofilm growth on solid supports. A recent review, which detail the pros and cons of current quantitative and qualitative biofilm characterisation methods [48] points out that biofilms must be homogenized in order to generate disperse cells in liquid media prior to analysis. However, disruptive methods cannot provide details on the biological, chemical, and physical properties within the native architecture of the biofilm, nor can they visualize in real-time the development of biofilms. The optotracer-based biofilm method overcomes these limitations, as optotracers in the agar continuously report the state of the biofilm via monitoring of the ECM. Combined with GFP-expressing bacteria, the relation between metabolically active cells and the spatial-temporal dynamics of the ECM composition and structural features can be analysed.

Our non-disruptive method showed that the inoculum placed on the agar surface may consist of bacteria harvested from exponential or stationary phase cultures, neither growth phase influenced the morphology of the growing biofilm. Bacterial growth, detected by GFP fluorescence in the automated microscope, is most pronounced at the edge of the inoculum. Spectrophotometric real-time recording of GFP fluorescence at this location showed an apparent lag phase, followed by an exponential increase that reaches a plateau resembling a stationary phase. This coincided with the start of ECM formation, shown by increased fluorescence from the Ebba680 optotracer. This staggered system by which ECM formation closely follows bacteria growth was observed in all regions of the biofilm, suggesting that radial expansion of the biofilm occurs in a phasic pattern similar to that of surface attached biofilms observed in our previous work [5]. At later timepoints, biofilm expansion is eventually arrested, as the biofilm by an unknown mechanism senses a limitation of available space. Detection of channel-like structures and unique regions in the biofilm formed by radially organized ECM demonstrates the phenomenon of compartmentalization, which substantiates the hypothesis of differentiation of roles within biofilms [49,50]. By establishing functionally specialized cells, bacteria are able to withstand the challenges imposed from a wider range of natural environments and man-made treatments [51].

To minimize the need for manual analysis, the semi-high throughput biofilm method presented here utilized automated microscopy and spectroscopy. As these instruments did not possess the capability to view the vertical stratification of the ECM, our assessment focused on horizontal coordinates. Despite this limitation, several similarities were observed between this study and others. The ridges rich with ECM in this study appear similar to those generated when the outer zone of the biofilm buck and rise [52]. The vertical folding of dense once flat colonies into double layers, whereby curli was observed to exclusively stack in the top layer, is likely to be involved in the formation of the channel-like structures observed in our study. Furthermore, the non-uniform distribution of curli we observed when performing confocal microscopy from the bottom of the wt biofilm is consistent with previously reported patchy curli expression in deeper regions of *E. coli* biofilms [18]. Also the phasic pattern of bacterial growth and curli formation is consistent with previous reports on the spatial-temporal sequence of physiological changes during *E. coli* biofilm formation, with curli expression beginning in the post‐exponential phase [18,49]. However, absence of curli ECM within the center of the wt-p2777 biofilm was contrary to previous findings showing that a high-level curli production is a prerequisite for the formation of wrinkles and ring-like microcolonies [20,49]. Whether this is due to reduced dimensions of the colonizable agar, impacting the vertical development of the biofilm, is currently unknown.

## 5 Conclusion

Based on the unique photophysics of a new series of dynamic fluorescent tracer molecules, a method is presented that enables real-time analysis of biofilm formation on solid supports. By selective tracing of ECM components, the method allows definitive biofilm monitoring, thereby reducing the inherent biases of morphotyping. The use of optotracing to study the formation of amyloid fibers in other bacterial species is of interest and under development. We envision that the semi-high throughput method will increase the ease and efficiency of biofilm detection, and will generate deeper understanding of the impact of biofilm on bacterial colonization and infection at air-solid interfaces.

## CRediT authorship contribution statement

**Ferdinand X. Choong:** Conceptualization, Methodology, Validation, Formal analysis, Writing – original draft, Visualization, Supervision, Project administration. **Smilla Huzell:** Formal analysis, Investigation. **Ming Rosenberg:** Validation, Investigation. **Johannes A. Eckert:** Validation, Investigation. **Madhu Nagaraj:** Investigation, Resources. **Tianqi Zhang:** Investigation. **Keira Melican:** Investigation, Supervision. **Daniel E. Otzen:** Conceptualization, Methodology, Resources, Writing – review & editing, Supervision, Project administration, Funding acquisition. **Agneta Richter-Dahlfors:** Conceptualization, Methodology, Resources, Writing – review & editing, Visualization, Supervision, Project administration, Funding acquisition.

## Declaration of competing interest

The authors declare the following financial interests/personal relationships which may be considered as potential competing interests: Agneta Richter-Dahlfors reports a relationship with Ebba Biotech AB that includes: board membership and equity or stocks. Ferdinand X. Choong reports a relationship with Ebba Biotech AB that includes: equity or stocks. Agneta Richter-Dahlfors has patent issued to Richter Life Science Development AB. Agneta Richter-Dahlfors has patent pending to Richter Life Science Development AB. Ferdinand X. Choong has patent issued to Richter Life Science Development AB. Ferdinand X. Choong has patent pending to Richter Life Science Development AB. Co-author (S.H.) became employed by Ebba Biotech a year after she participated in the study as a summer student.
