# Supplementary material for: A semi high-throughput method for real-time monitoring of curli producing Salmonella biofilms on air-solid interfaces
Source: Biofilm. 2021 Nov 13;3:100060. doi: 10.1016/j.bioflm.2021.100060 (PMC8605384; doi:10.1016/j.bioflm.2021.100060)
Supplement: Multimedia component 2 [file mmc2.docx]

**SUPPLEMENTARY INFORMATION**

**A semi high-throughput method for real-time monitoring of curli producing *Salmonella* biofilms on air-solid interfaces**

Ferdinand X. Choong^a,b,^*, Smilla Huzell^a,b,#^, Ming Rosenberg^a,b^, Johannes A. Eckert^a,b,†^, Madhu Nagaraj^c^, Tianqi Zhang^a,b^, Keira Melican^a,b^, Daniel E. Otzen^c^,

Agneta Richter-Dahlfors^a,b,^*

^a^ AIMES - Center for the Advancement of Integrated Medical and Engineering Sciences at Karolinska Institutet and KTH Royal Institute of Technology, Stockholm, Sweden

^b^ Department of Neuroscience, Karolinska Institutet, Stockholm, Sweden

^c^ iNANO and Department of Molecular Biology and Genetics, Aarhus University, Aarhus, Denmark

^#^ Present address: Ebba Biotech AB, Stockholm, Sweden

† Present address: Department of Biology, ETH Zurich, Schweiz

* Corresponding authors

Ferdinand X. Choong, [xiankeng.choong@ki.se](mailto:xiankeng.choong@ki.se), Phone: +46 73 8412454

Agneta Richter-Dahlfors, [agneta.richter.dahlfors@ki.se](mailto:agneta.richter.dahlfors@ki.se), Phone: +46 70 2577425

**Page index**

Supplementary Table 1 *Strains and plasmids used in this study*……………………...……..2

Supplementary Figure 1 *Calcofluor assay showing cellulose production*………...……..........3

Supplementary Figure 2 *Automated microscopy showing the morphotypes of biofilm*

*macrocolonies in the presence of Ampicillin*…..................................4

Supplementary Figure 3 *Grid pattern for selective spectral area scans*…………...................4

Supplementary Video 1 *Formation of the macrocolony biofilm in real-time*…………….…..5

**Supplementary Table 1**

***Strains and plasmids used in this study***

|  |  |  | | |  | |  |
| --- | --- | --- | --- | --- | --- | --- | --- |
| **Strains or Plasmids** |  | **Characteristics** | | **Source** | | | |
| **Strains** | **Alternate name** |  |  | | |  |  |
| 3934 | wt | Wild-type clinical isolate | | Solano, C. et al. 2002 ^35^ | | | |
| 3934*ΔcsgD* | *ΔcsgD* | 3934*ΔcsgD*::KmR | | García et al. 2004 ^36^ | | | |
| 3934*ΔcsgA* | *ΔcsgA* | 3934*ΔcsgA*::KmR | | Latasa et al. 2005 ^37^ | | | |
| 3934*ΔbcsA* | *ΔbcsA* | 3934*ΔbcsA*::KmR | | Solano, C. et al. 2002 ^35^ | | | |
| 3934 p2777 | wt-p2777 | 3934 expressing green fluorescence protein, AmpR, KmR | | Choong, F.X. et al. 2016 ^22^ | | | |
| 3934*ΔcsgD* p2777 | *ΔcsgD*-p2777 | 3934*ΔcsgD* expressing green fluorescence protein, AmpR, KmR | | Choong, F.X. et al. 2016 ^22^ | | | |
| 3934*ΔcsgA* p2777 | *ΔcsgA*-p2777 | 3934*ΔcsgA* expressing green fluorescence protein, AmpR, KmR | | Choong, F.X. et al. 2016 ^22^ | | | |
| 3934*ΔbcsA* p2777 | *ΔbcsA*-p2777 | 3934*ΔbcsA* expressing green fluorescence protein, AmpR, KmR | | Choong, F.X. et al. 2016 ^22^ | | | |
| UPEC 12 |  | Wild-type clinical uropathogenic isolate | | Kai-Larsen, Y. etal 2010 [[38]](https://paperpile.com/c/E50CKs/H7sEn) | | | |
| **Plasmids** |  |  | |  | | | |
| p2777 |  | Vector for green fluorescent protein expression and ampicillin resistance | | Hautefort, I. et al. 2003 ^38^ | | | |
| pFPV25.1* |  | Vector for green fluorescent protein expression and ampicillin resistance | | Valdivia, Falcow 1996 ^41^ | | | |
|  |  |  | |  | | | |

*pFPV25.1 was a gift from Raphael Valdivia (Addgene plasmid #20668; <http://n2t.net/addgene:20668>;

RRID: Addgene_20668)

**References**

Choong, F. X., Bäck, M., Fahlén, S., Johansson, L. B., Melican, K., Rhen, M., et al. (2016a). Real-time optotracing of curli and cellulose in live biofilms using luminescent oligothiophenes. *NPJ Biofilms Microbiomes* 2, 16024.

Solano, C., García, B., Valle, J., Berasain, C., Ghigo, J.-M., Gamazo, C., et al. (2002). Genetic analysis ofSalmonella enteritidisbiofilm formation: critical role of cellulose. *Molecular Microbiology* 43, 793–808. doi:10.1046/j.1365-2958.2002.02802.x

García, B., Latasa, C., Solano, C., Portillo, F. G., Gamazo, C., and Lasa, I. (2004). Role of the GGDEF protein family in Salmonella cellulose biosynthesis and biofilm formation. *Molecular Microbiology* 54, 264–277. doi:10.1111/j.1365-2958.2004.04269.x.

Kai-Larsen Y, Lüthje P, Chromek M, Peters V, Wang X, Holm A, et al. Uropathogenic Escherichia coli modulates immune responses and its curli fimbriae interact with the antimicrobial peptide LL-37. PLoS Pathog 2010;6:e1001010.

Valdivia RH, Falcow S. Bacterial genetics by flow cytometry: rapid isolation of Salmonella typhimurium acid-inducible promoters by differential fluorescence induction. Mol Micro. 1996 Oct. 22(2):367-78. 10.1046/j.1365-2958.1996.00120.x

**Supplementary Figure 1**

***Calcofluor assay showing cellulose production***

Automated microscopy shows biofilm macrocolonies formed in the 6-well plate Calcofluor assay, in which bacteria grow on agar supplemented with Calcofluor White. Morphologies of the isogenic collection of strains **(A)** wt (curli+, cellulose +), **(B)** *ΔbcsA* (curli+, cellulose-), (**C**) *ΔcsgA* (curli-, cellulose+), and (**D**) *ΔcsgD* (curli-, cellulose-) are shown in brightfield images, while fluorescence microscopy shows the spatial distribution of calcofluor stained cellulose separately and in merged images of respective strain. Scale bar = 5 mm.


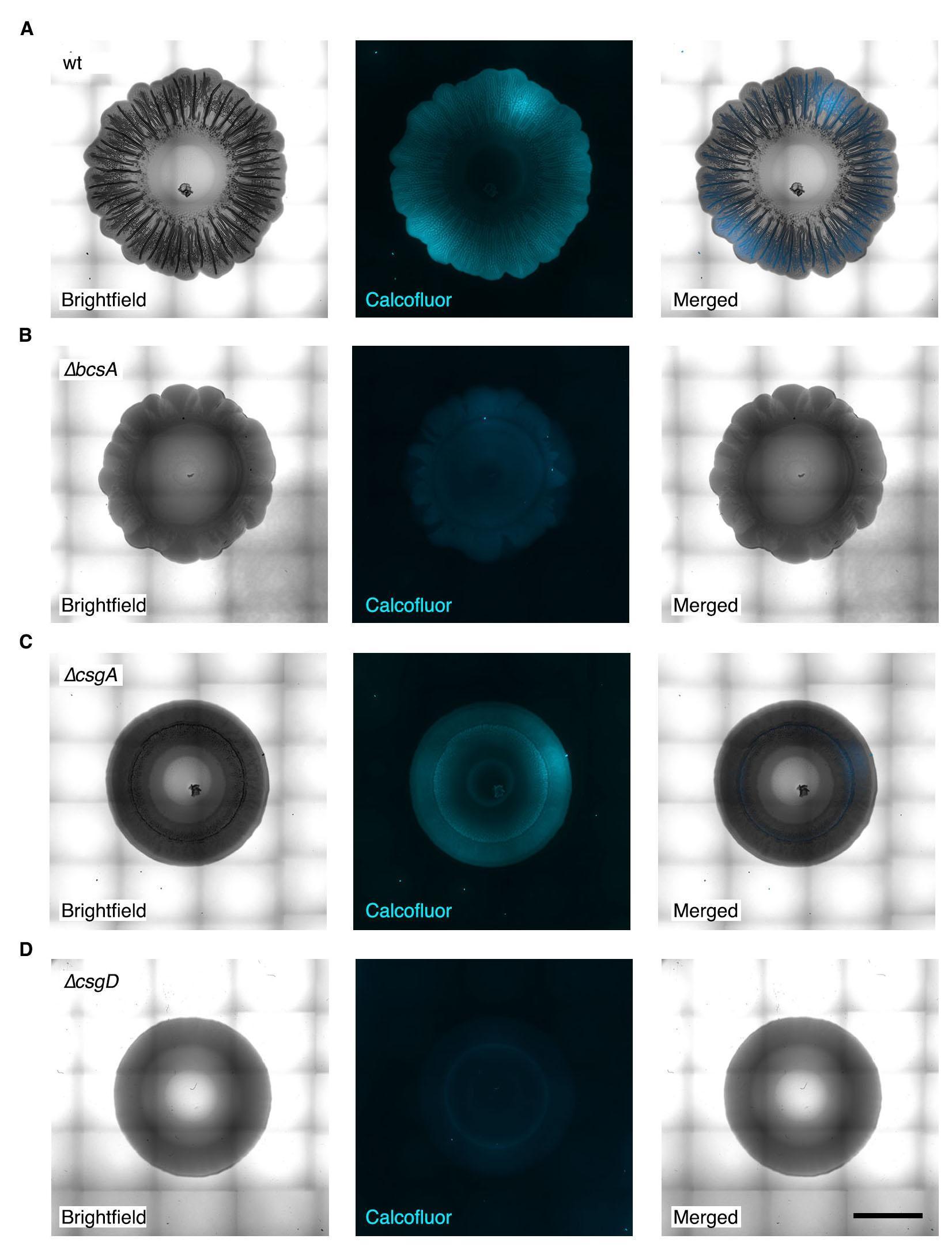


**Supplementary Figure 2**

***Automated microscopy showing the morphotypes of biofilm macrocolonies in the presence of Ampicillin***

Automated microscopy shows biofilm macrocolonies formed in the 6-well plate optotracing assay, in which bacteria grow on agar supplemented with EbbaBiolight 680 (Ebba680). Morphologies of the GFP-expressing strain wt-p2777 (curli+, cellulose+) shown by brightfield and fluorescence microscopy. GFP channel shows the spatial distribution of bacteria (green). Red channel shows curli ECM stained by Ebba680 (red). Ampicillin (100 μg/ml) was added to the agar to ensure that bacteria maintained the GFP-encoding plasmid. Scale bar = 10 mm.


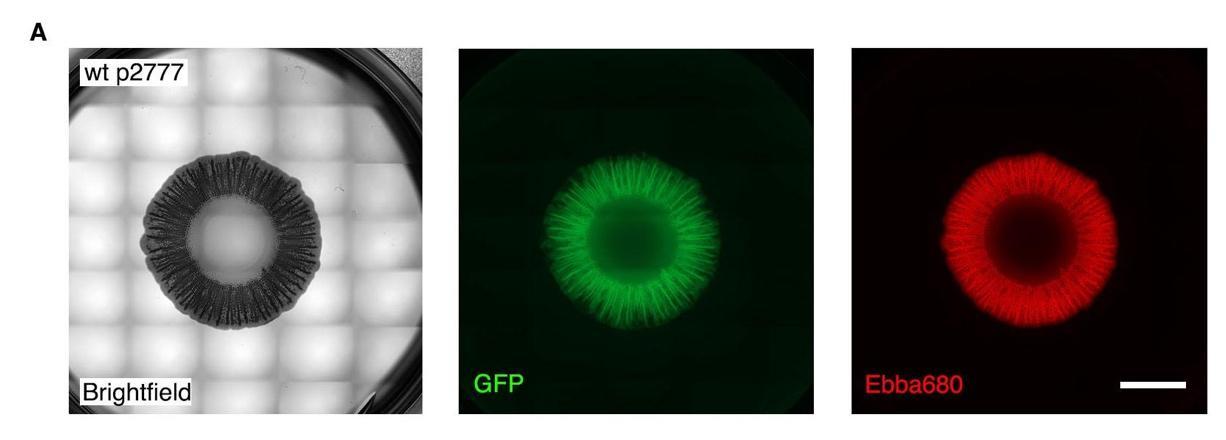


**Supplementary Figure 3**

***Grid pattern for selective spectral area scans***

Schematic illustration of the grid pattern used by the microplate reader to enable spectral scans in selected areas of each well of a 6-well plate. With a central location of the inoculum on the agar supplemented by EbbaBiolight 680, we utilized the 15x15 area scan mode (circle-filled function) of the software in order to collect spectra in the areas H8-H11 as the biofilm formed (see **Figure 6B-D** in main text).

**
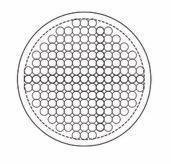
**

**Supplementary Video 1**

***Monitoring the formation of the macrocolony biofilm in real-time***

Fluorescence monitoring of *Salmonella* biofilm formation in real-time is achieved by growing wt-p2777 on agar supplemented with EbbaBiolight 680 in a 6-well plate, which is incubated in a Lionheart™ FX Automated Microscope. Bacterial growth is monitored by bacterial GFP-expression (green). Curli fibrils (red) are visualized when bacteria express and secrete this ECM component, making it available as a binding target for EbbaBiolight 680. The video shows the spatial-temporal development of a macrocolony grown at 28°C for 60 h, with images collected at 2 h intervals.
